# Hematopoietic stem progenitor cells with malignancy‐related gene mutations in patients with acquired aplastic anemia are characterized by the increased expression of CXCR4

**DOI:** 10.1002/jha2.515

**Published:** 2022-07-03

**Authors:** Takamasa Katagiri, Jorge Luis Espinoza, Mizuho Uemori, Honoka Ikeda, Kohei Hosokawa, Ken Ishiyama, Takeshi Yoroidaka, Tatsuya Imi, Hiroyuki Takamatsu, Tatsuhiko Ozawa, Hiroyuki Kishi, Yasuhiko Yamamoto, Mahmoud Ibrahim Elbadry, Yoshinori Yoshida, Kazuhisa Chonabayashi, Katsuto Takenaka, Koichi Akashi, Yasuhito Nannya, Seishi Ogawa, Shinji Nakao

**Affiliations:** ^1^ Department of Clinical Laboratory Science Graduate School of Medical Science Institute of Medical Pharmaceutical and Health Sciences Kanazawa University Kanazawa Ishikawa Japan; ^2^ Department of Occupational Therapy Graduate School of Medical Science Institute of Medical Pharmaceutical and Health Sciences Kanazawa University Kanazawa Ishikawa Japan; ^3^ Department of Hematology Faculty of Medicine Institute of Medical Pharmaceutical and Health Sciences Kanazawa University Kanazawa Ishikawa Japan; ^4^ Department of Immunology Faculty of Medicine Academic Assembly University of Toyama Toyama City Toyama Japan; ^5^ Department of Biochemistry and Molecular Vascular Biology Kanazawa University Graduate School of Medical Sciences Kanazawa Ishikawa Japan; ^6^ Division of Hematology Department of Internal Medicine Faculty of Medicine Sohag University Sohag Egypt; ^7^ Center for iPS Cell Research and Application Kyoto University Sakyo‐ku Kyoto Japan; ^8^ Department of Hematology and Oncology Graduate School of Medicine Kyoto University Sakyo‐ku Kyoto Japan; ^9^ Department of Hematology Clinical Immunology and Infectious Diseases Ehime University Graduate School of Medicine Toon Ehime Japan; ^10^ Department of Medicine and Biosystemic Science Kyushu University Graduate School of Medical Sciences Fukuoka City Fukuoka Japan; ^11^ Division of Hematopoietic Disease Control Institute of Medical Science University of Tokyo Minato‐ku Tokyo Japan; ^12^ Department of Pathology and Tumor Biology Kyoto University Yoshida‐Konoe‐cho Sakyo‐ku Kyoto Japan; ^13^ Institute for the Advanced Study of Human Biology (WPI‐ASHBi) Kyoto University Sakyo‐ku Kyoto Japan; ^14^ Department of Medicine Centre for Hematology and Regenerative Medicine Karolinska Institute Stockholm Sweden

**Keywords:** acquire aplastic anemia, clonal hematopoiesis, CXCR4, hematopoietic stem progenitor cell (HSPC), HLA class I allele‐lacking cell

## Abstract

The phenotypic changes in hematopoietic stem progenitor cells (HSPCs) with somatic mutations of malignancy‐related genes in patients with acquired aplastic anemia (AA) are poorly understood. As our initial study showed increased CXCR4 expression on HLA allele‐lacking (HLA[−]) HSPCs that solely support hematopoiesis in comparison to redundant HLA(+) HSPCs in AA patients, we screened the HSPCs of patients with various types of bone marrow (BM) failure to investigate their CXCR4 expression. In comparison to healthy individuals (*n* = 15, 12.3%–49.9%, median 43.2%), the median CXCR4^+^ cell percentages in the HSPCs of patients without somatic mutations were low: 29.3% (14.3%–37.3%) in the eight patients without HLA(−) granulocytes, 8.8% (4.1%–9.8%) in the five patients with HLA(−) cells accounting for >90% of granulocytes, and 7.8 (2.1%–8.7%) in the six patients with paroxysmal nocturnal hemoglobinuria. In contrast, the median percentage was much higher (78% [61.4%–88.7%]) in the five AA patients without HLA(−) granulocytes possessing somatic mutations (*c‐kit*, t[8;21], monosomy 7 [one for each], *ASXL1* [*n* = 2]), findings that were comparable to those (66.5%, 63.1%–88.9%) in the four patients with advanced myelodysplastic syndromes. The increased expression of CXCR4 may therefore reflect intrinsic abnormalities of HSPCs caused by somatic mutations that allow them to evade restriction by BM stromal cells.

## 1 INTRODUCTION

Acquired aplastic anemia (AA) is a syndrome characterized by pancytopenia and bone marrow (BM) hypoplasia without apparent dysplasia in the BM cells and an increase in the number of blasts [1]. As the main pathophysiology of AA is a reduction of hematopoietic stem progenitor cells (HSPCs) due to T‐cell attacks against HSPCs, HSPCs themselves are thought to be healthy and give rise to polyclonal hematopoiesis [2, 3, 4, 5]. However, recent studies using next‐generation sequencing have revealed that approximately one third of newly diagnosed AA patients have clonal hematopoiesis by HSPCs with somatic mutations of myeloid malignancy‐related genes [6, 7]. Some of these somatic mutations indeed predispose secondary myelodysplastic syndromes (MDS) [1, 8, 9], whereas others do not directly contribute to the development of secondary MDS [6]. The prognostic significance of such somatic mutations therefore remains unclear. Phenotypic changes in the HSPCs associated with somatic mutations are unknown either.

Hematopoiesis in humans is thought to be supported by a limited number of HSPCs selected from a large number of HSPCs that remain dormant in the BM [10, 11]. Different from murine HSPCs that can be marked by transduced genes, it is generally impossible to know which HSPCs do or do not contribute to hematopoiesis due to a lack of useful markers on healthy HSPCs, which are responsible for active hematopoiesis. Rare exceptions include HLA class I allele‐lacking (HLA[−]) HSPCs, which are detected in approximately 30% of AA patients [4, 5, 12]. HLA(−) HSPCs are thought to be healthy HSPCs that escape T‐cell attack due to the lack of particular HLA‐class I alleles that present autoantigens of HSPCs to T cells in AA [13, 14]. As HLA(−) HSPCs often produce clonal hematopoiesis with their HLA(−) progenies, studying HLA(−) HSPCs may be useful for clarifying the phenotype unique to healthy HSPCs, which substantially contribute to hematopoiesis and could be different from the phenotype of HSPCs with myeloid malignancy‐related gene mutations.

To address this issue, we investigated the expression of the HLA alleles in the HSPCs of AA patients whose granulocytes are completely replaced with HLA(−) cells. In the process of studying the gene expression profiles in HLA(−) HSPCs and HLA(+) HSPCs of an AA patient, we found that the gene expression of a chemokine *CXCL12* was diminished in HLA(−) HSPCs, and that the expression of its receptor CXCR4 on HSPCs was also decreased in comparison to HLA(+) HSPCs. As the CXCR4 expression on HSPCs has been shown to increase in patients with MDS, we hypothesized that the CXCR4 expression level may be useful for discriminating clonal hematopoiesis by healthy HSPCs from premalignant clonal hematopoiesis by HSPCs with somatic mutations. We then screened HSPCs from the peripheral blood (PB) of patients with various types of BM failure with or without somatic mutations of myeloid malignancy‐related genes for the expression of CXCR4.

## 2 MATERIALS AND METHODS

### 2.1 Patients

Patient information is provided in Table S1. All patients and healthy volunteers provided their informed consent in accordance with the Declaration of Helsinki. This study was approved by the Ethics Committee of Kanazawa University Institute of Medical, Pharmaceutical, and Health Sciences (nos. 2016‐284 and 2018‐017). All data generated or analyzed during this study are included in this article and the Supporting Information section. The data described in this article are openly available.

### 2.2 Flow cytometry analysis and cell sorting

Details of methods are provided in Table S2 and Figure S1.

### 2.3 Microarray analysis

HLA(−) and HLA(+)common myeloid progenitors (CMPs) from AA patients as well as CMPs from healthy BM cells were subjected to gene expression analyses using a whole human genome Oligo Microarrays Ver2.0 (4 × 44K, G2519F#26652, Agilent Technologies). In brief, RNA was extracted with RNeasy Micro Kit (QIAGEN) from FACS‐sorted HLA(−) CMPs, HLA(+) CMPs, and normal CMPs of healthy BM cells, according to the manufacturer's guidelines. The quality of the purified RNA was verified using an Agilent TapeStation 2200 (Agilent Technologies). Fluorescent cyanine‐3‐cytidine triphosphate‐labeled cRNA was used for hybridization to human oligo microarray slides (Agilent Technologies) for 17 h at 65°C. The hybridized microarray slides were washed according to the manufacturer's instructions. Intensity values of each scanned feature were quantified using Agilent feature extraction software 11.0.1.1 that performed background subtractions. We only used features that were flagged as no errors (detected flags) and excluded features that were not positive, not significant, not uniform, not above background, saturated, and population outliers (not detected and compromised flags). Normalization was performed using Agilent GeneSpring software version 12.6 (per chip: normalization to Quantile, per gene: normalization to the median of all samples). To investigate gene expression differences, we selected genes that were up‐ or downregulated by setting a threshold of twofold with <0.01 of a cut‐off *p‐*value (Table S3, the details of methods are provided in the Supporting Information section).

### 2.4 Generation of iPS cells (iPSCs)

Heparinized PB was drawn from an AA patient (UPN 2) in convalescence with HLA(−) leukocytes of which hematopoietic function depended on cyclosporine for 14 years after anti‐thymocyte globulin therapy and induced pluripotent stem cells (iPSCs) were generated from the circulating monocytes at the Center for iPS Cell Research and Application (Kyoto University, Kyoto, Japan) as described previously [14]. Several clones with either HLA(−) or HLA(+) phenotype were obtained and verified as previously reported [14]. Maintenance and expansion of iPSCs were achieved by coculturing them with mitomycin C‐treated SNL feeder cells in Dulbecco's‐modified Eagle medium/F12 supplemented with 20% knockout serum replacement medium, fibroblast growth factor (10 ng/ml), 2‐mM l‐glutamine, nonessential amino acids, and 1% penicillin and streptomycin.

### 2.5 Induction of HSPCs from iPSCs

CD34^+^ HSPCs derived from iPSCs were generated by culture in the conditioned medium (CM) derived from OP9 cells and WEHI cells and were resuspended in phosphate‐buffered saline (PBS) containing 1% of bovine serum albumin and stained with various monoclonal antibodies (mAbs) directed against iPSCs and hematopoietic cell markers (Table S2) [14]. In some experiments, CD34^+^ HSPCs derived from iPSCs were purified by cell sorting using a FACSAria Fusion instrument and were then subjected to colony‐forming assays using methylcellulose medium (MethoCult GF H4034; STEMCELL Technologies, Vancouver, BC, Canada). Enriched CD34^+^ HSPCs derived from iPSCs were resuspended at the density of 50,000 cells per 1 mL. An amount of 200 µl of the cell suspension was mixed with 1‐ml methylcellulose medium, and then plated into a 35‐mm dish and incubated at 37°C with 5% CO_2_ for 2 weeks. Lineage classification was identified by the morphologic analysis of Giemsa staining with an inverted microscope.

### 2.6 Transplantation of human CD34^+^ iPSC‐HSPCs cells into immunodeficient mice

The immunodeficient mouse strain C57BL/6.*Rag2* ^null^ *Il2rg* ^null^NOD*Sirpa* (BRGS), which lacks T, B, and NK cells, was used as a humanized model of human hematopoiesis. Sublethally irradiated (250 cGy) mice of 4 weeks of age received a total of 1 × 10^5^ CD34^+^ HSPCs derived from iPSCs by direct injection into the BM of femur bones. At 12 weeks after transplantation, animals were euthanized and spleen was harvested from recipient mice and subjected to flow cytometry analysis as described earlier [14].

### 2.7 Analysis of somatic mutations in PB leukocytes

PB samples from AA and MDS patients were subjected to DNA extraction using a DNA extraction kit (Qiagen, Hilden, Germany). The DNA samples were subjected to targeted‐capture sequencing as previously described [15]. A custom RNA bait was designed for the detection of oncogenic variants in 390 known driver genes implicated in myeloid malignancies (SureSelect; Agilent Technology). This bait included additional 1158 single‐nucleotide polymorphisms to calculate genome‐wide copy numbers. These probes were deliberately selected so that they cover the human genome uniformly to allow for prospective detection of copy‐number change and loss of heterozygosity (LOH) on the next‐generation sequencing platform.

### 2.8 Statistical analyses

Data were analyzed using the Spearman's correlation using the EZR software package [16] and the GraphPad Prism software package, version 5.02 (San Diego, CA), and values were shown as the mean of the individual sample ± standard error of the mean. Findings were judged to be statistically significant if *p*‐values were <0.05.

## 3 RESULTS

### 3.1 The percentages of HLA(−) cells in different CD34^+^ cell subsets, showing lower percentages of HLA(−) cells in CMPs in comparison to MEPs and GMPs

**FIGURE 1:**
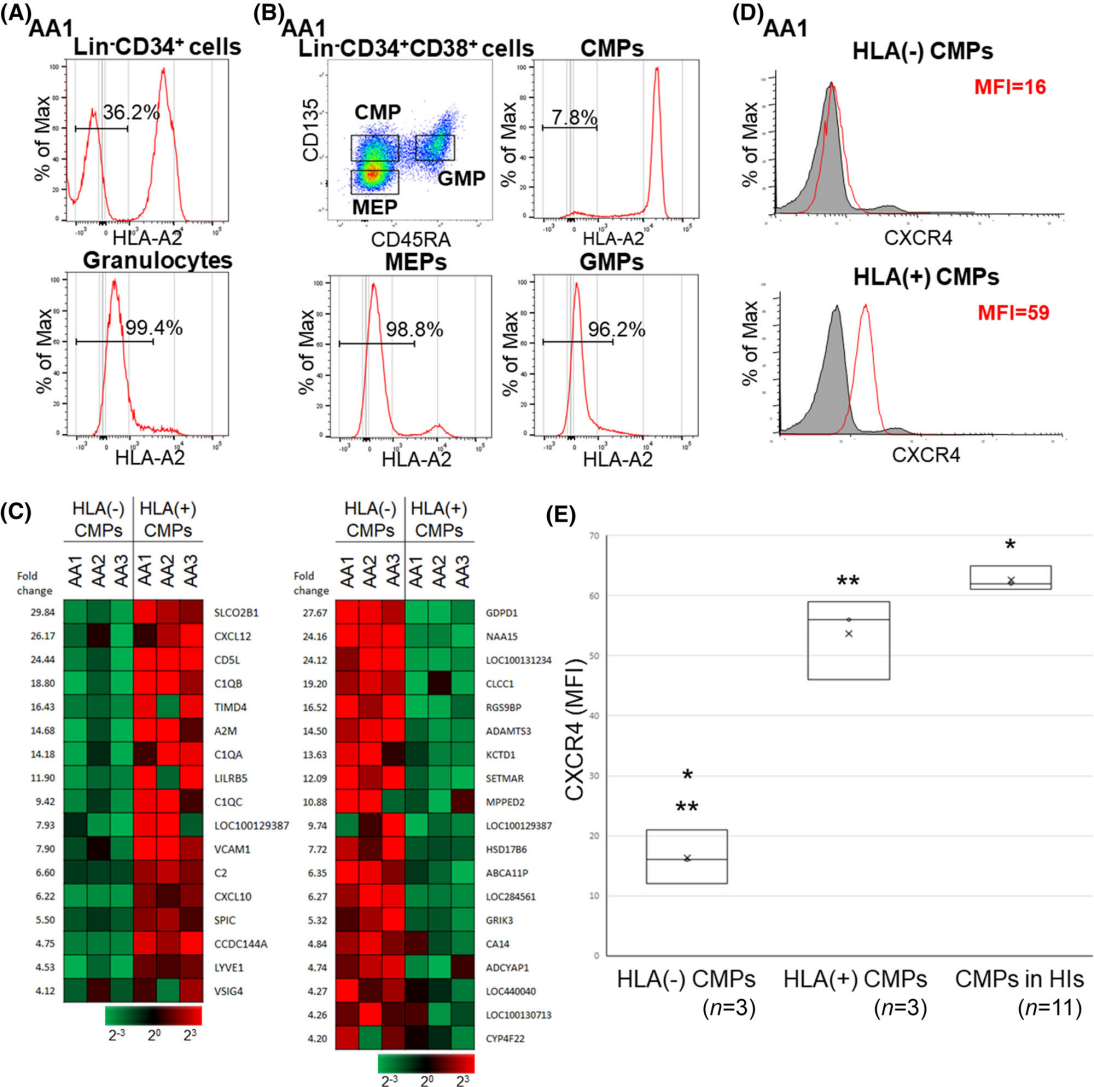
Distinctive features of HLA(−) hematopoietic stem progenitor cells (HSPCs) in patients with aplastic anemia (AA). (A) The percentages of HLA(−) cells in bone marrow (BM) lineage (Lin)^−^CD34^+^ cells and peripheral blood (PB) granulocytes of a patient with AA (AA1). (B) The percentages of HLA(−) cells in different CD34^+^ cell subsets of the same patient (AA1), showing lower percentages of HLA(−) cells in common myeloid progenitors (CMPs) in comparison to myeloid‐erythroid progenitors (MEPs) and granulocyte‐macrophage progenitors (GMPs). (C) Differences in the gene expression of HLA(−) and HLA(+) CMPs from three AA patients. Clusters of genes with expression that were higher in HLA(+) CMPs (left) and HLA(−) CMPs (right) are shown. (D) The CXCR4 expression on HLA(−) CMPs and HLA(+) CMPs of an AA patient (AA1) and on CMPs of a healthy individual (HI) (HI1). The percentage shows the CXCR4 expression of the AA patient. (E) The expression of CXCR4 by HLA(−) and HLA(+) cells in CMPs of three AA patients and HIs. MFI, mean fluorescence intensity. ^*^ *p *< 0.05. ^**^ *p *< 0.05

We characterized HSPCs in the BM of three AA patients (AA1‐3) in remission whose HLA(−) granulocytes accounted for 60.3%–99.4% of the total granulocytes and for whom BM cells were available. Percentages of HLA(−) cells were low at 29.7%–39.9% of the total lineage (Lin)^−^CD34^+^ cells (Figures 1A and S2) and much lower in common myeloid progenitors (CMPs, 4.5%–12.9%) than in megakaryocyte‐erythroid progenitors (MEPs, 69.9%–98.8%) and granulocyte‐macrophage progenitors (GMPs, 96.2%–97.8%) of respective patients (Figures 1B and S3), suggesting that only some primitive HSPCs substantially contribute to hematopoiesis in these patients.

### 3.2 The expression of CXCR4 by HLA(−) cells in CMPs of AA patients was lower than that of HLA(+) cells in CMPs

Microarray analyses of sorted HLA(−) and HLA(+) CMPs revealed that the expression of several chemokine genes, including *CXCL12*, was diminished in HLA(−) CMPs (Figure 1C). The reduced expression of the *CXCL12* gene in HLA(−) CMPs compared with HLA(+) CMPs prompted an investigation of the expression of *CXCL12* receptor CXCR4 on CMPs and other progenitor cell subsets, as *CXCL12* excreted by HSPCs reportedly augments the expression of CXCR4 by itself [17, 18]. The CXCR4 expression by HLA(−) CMPs was much lower than in the HLA(+) counterparts in all three patients (Figure 1D). The percentages of CXCR4(+) cells (1.7%–3.6%) in HLA(−) CMPs were significantly lower than in HLA(+) CMPs (11.5%–24.6%, *p *< 1.0 × 10^−3^) and healthy individuals (HIs) (*n* = 11, 28.5%–32.1%, *p *< 1.0 × 10^−3^) (Figure 1E). The CXCR4 expression on MEPs and GMPs, which consisted of almost entirely HLA(−) cells, was also lower than in HIs (data not shown).

### 3.3 The CXCR4 expression on CD34^+^ HSPCs induced from iPSC clones with different genotypes

**FIGURE 2:**
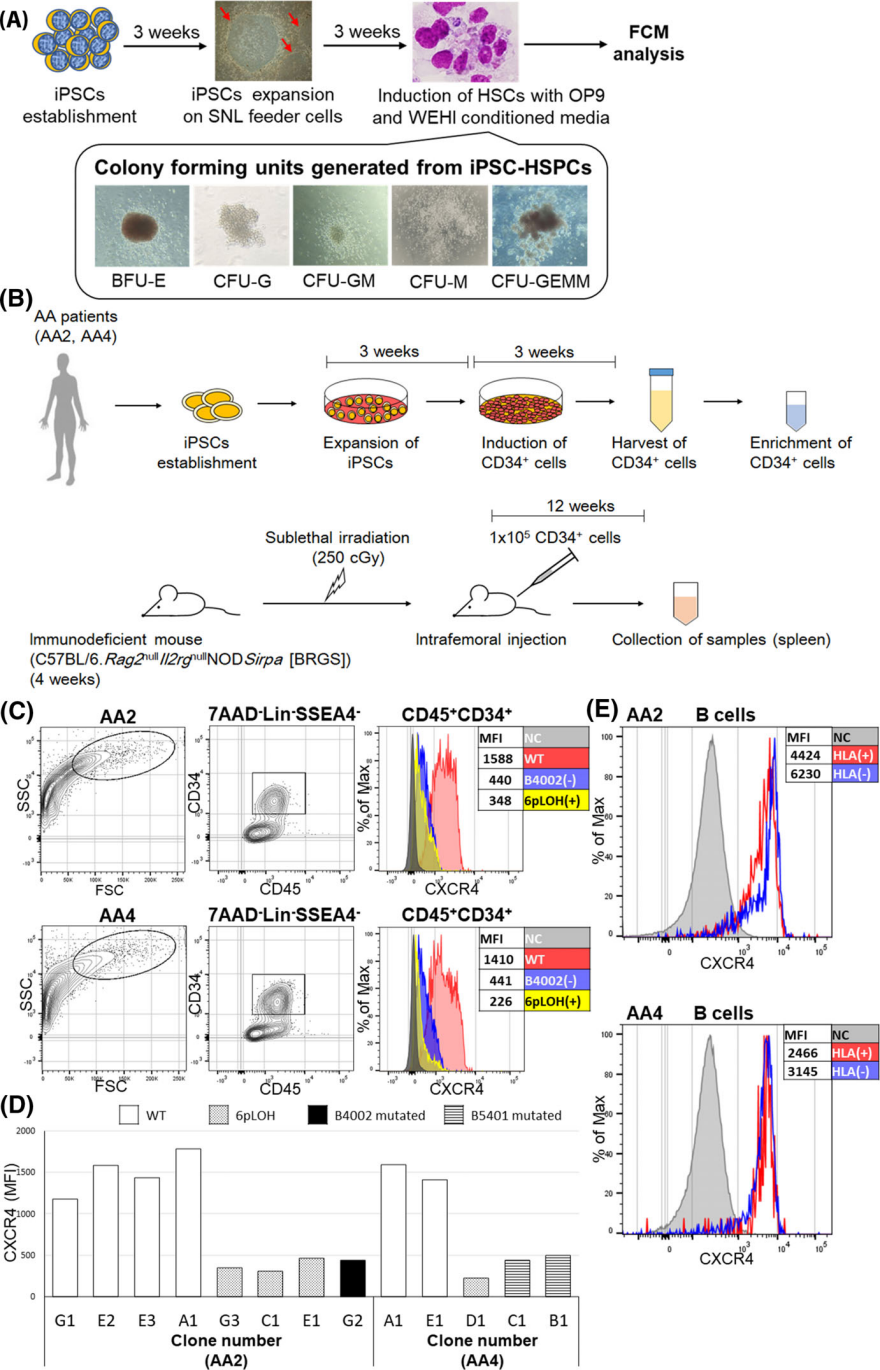
A schematic diagram for the induction of CD34^+^ cells from induced pluripotent stem cells (iPSCs) and intrafemoral injection of the iPSC‐hematopoietic stem progenitor cells (HSPCs) into BRGS mice and the CXCR4 expression on CD34^+^ cells induced from iPSC clones with different genotypes. (A) Incubation of iPSCs in conditioned medium induced CD34^+^ cells capable of generating different types of colony‐forming cells. Red arrows indicate iPSCs expanded on SNL feeder cells. (B) Sublethally irradiated (250 cGy) BRGS mice of 4 weeks of age received a total of 1 × 105 CD34+ HSPCs derived from iPSCs by direct injection into the bone marrow (BM) of femur bones. At 12 weeks after transplantation, animals were euthanized and spleen was harvested from recipient mice and subjected to flow cytometry (FCM) analysis. (C) The gating strategy to identify CD34^+^ cells induced from iPSCs and the expression of CXCR4 on gated CD34^+^ cells that were HLA(+) (wild‐type [WT], in red), HLA‐B4002(−) in aplastic anemia (AA)2 and HLA‐B5401(−) in AA4 due to inactivating mutations in each HLA‐B allele (in blue), and HLA‐B(−) due to 6ploss of heterozygosity (LOH) (in yellow). (D) The CXCR4 expression on CD34^+^ cells induced from iPSC clones with different genotypes, showing lower expression in all HLA(−) CD34^+^ cells in comparison to WT CD34^+^ cells from both patients (AA2 carrying HLA‐B*40:02 and AA4 carrying HLA‐B*54:01). (E) The CXCR4 expression on B lymphocytes that were identified in the spleen of iPSC‐HSPC‐engrafted mice. The CXCR4 expression did not differ between WT and HLA(−) cells in both patients. BFU‐E, burst‐forming unit‐erythroid; BRGS C57BL/6.*Rag2* ^null^ *Il2rg* ^null^NOD*Sirpa;* CFU‐G, colony‐forming unit‐granulocyte; CFU‐GEMM, colony‐forming unit‐granulocyte, erythroid, macrophage, megakaryocyte; CFU‐GM, colony‐forming unit‐granulocyte/macrophage; CFU‐M, colony‐forming unit‐macrophage; Lin, lineage; MFI, mean fluorescence intensity

To ascertain the diminished CXCR4 expression on HLA(−) HSPCs in AA patients at the clonal level, we examined HSPCs derived from iPSCs with different genotypes, including wild‐type (WT), LOH of chromosome 6p (6pLOH), and a loss‐of‐function mutation in *HLA‐B* genes from one of the three patients (AA2) [14], and from another patient (AA4) who possessed HLA‐B5401‐lacking leukocytes [19]. Incubation of iPSCs in CM‐induced CD34^+^ cells capable of generating different types of colony‐forming cells (Figure 2A) as well as engrafting in immunodeficient mice (C57BL/6.*Rag2* ^null^ *Il2rg* ^null^NOD*Sirpa* [BRGS]), as previously described (Figure 2B) [14]. Both 6pLOH(+) CD45^+^CD34^+^ cells and HLA‐B (B4002 in AA2 and B5401 in AA4)‐lacking CD45^+^CD34^+^ cells expressed much less CXCR4 than WT cells (Figure 2C). Figure 2D summarizes the percentages of CXCR4^+^ cells in CD34^+^ cells induced from all iPSC clones with different genotypes, showing that the CXCR4 expression was consistently lower in HLA‐B(−) iPSC‐derived CD34^+^ cells than in WT cells in both patients. In contrast, B lymphocytes identified in the spleen of iPSC‐derived HSPC‐engrafted mice highly expressed CXCR4, regardless of the HLA expression (Figure 2E), suggesting that the CXCR4 expression reduction by iPSC‐HSPCs occurred by some epigenetic mechanism.

### 3.4 Comparison of the expression of CXCR4 between HLA(−) and HLA(+) or glycosylphosphatidylinositol‐anchored protein‐deficient (GPI[−]) and GPI(+) HSPCs in individual AA patients

**FIGURE 3:**
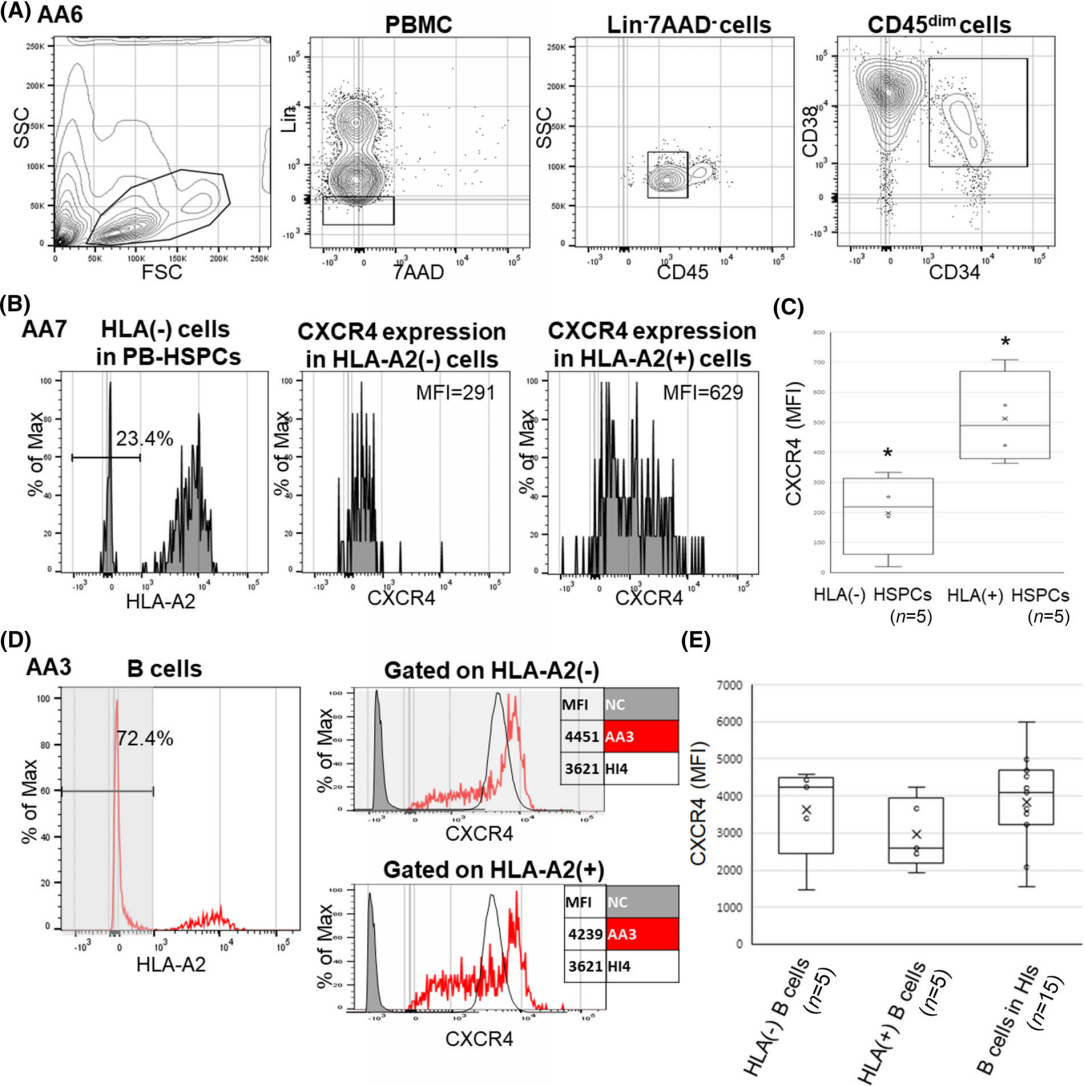
The gating strategy to detect CXCR4 on CD45^dim^CD34^+^CD38^+^ hematopoietic stem progenitor cells (HSPCs) in peripheral blood (PB) and CXCR4 expression by B cells from aplastic anemia (AA) patients possessing both HLA(−) and HLA(+) B‐cell populations. (A) Representative results of an AA6. (B) The CXCR4 expression on HLA(−) and HLA(+) HSPCs in patients whose HSPCs consisted of HLA(−) and HLA(+) cells (AA7). (C) The CXCR4 expression on HSPCs from five patients possessing both HLA(−) and HLA(+) cells. ^*^ *p *< 0.05. (D) CXCR4 expression levels by HLA(−) and HLA(+) B cells from an AA patient (AA3) are shown. (E) The graph summarizes CXCR4 expression levels by HLA(−) and HLA(+) B cells from five AA patients (AA2, 5–8) and HLA(+) B cells from 15 healthy individuals (HIs). Lin, lineage; MFI, mean fluorescence intensity; PBMC, peripheral blood mononuclear cell

**FIGURE 4:**
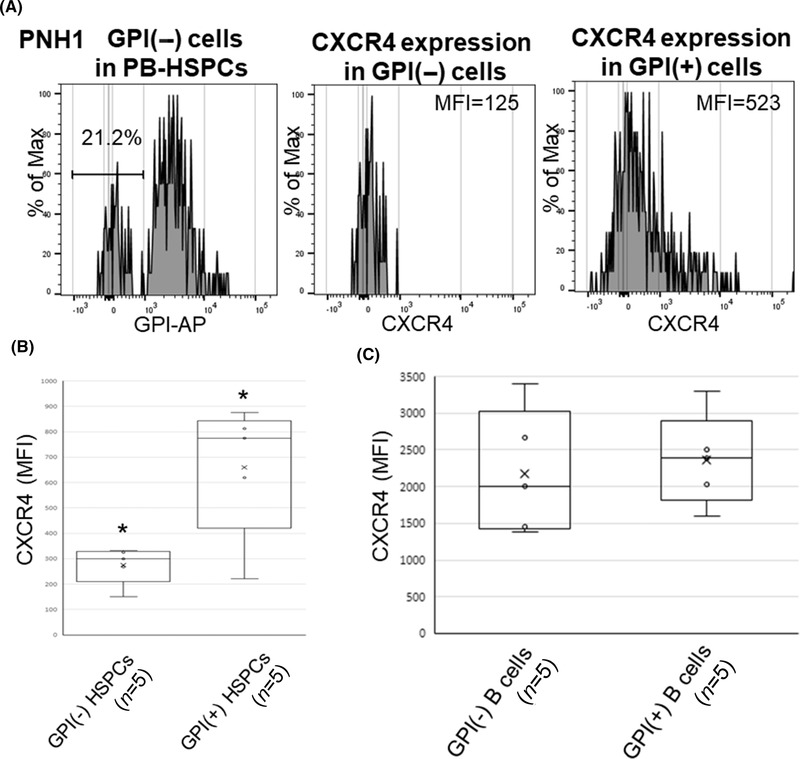
Comparison of the expression of CXCR4 between glycosylphosphatidylinositol (GPI)(−) cells and GPI(+)cells in patients with aplastic anemia (AA). (A) The CXCR4 expression on GPI(−) and GPI(+) hematopoietic stem progenitor cells (HSPCs) in patients whose HSPCs consisted of GPI(−) and GPI(+) cells (paroxysmal nocturnal hemoglobinuria [PNH]1). (B) The CXCR4 expression on HSPCs from five patients possessing both GPI(−) and GPI(+) cells. ^*^ *p *< 0.05. (C) The graph summarizes CXCR4 expression levels by GPI(−) and GPI(+) B cells from five PNH patients (PNH1–5). AP, anchored protein; MFI, mean fluorescence intensity; PB, peripheral blood

Four patients (AA5‐8) had both HLA(−) and HLA(+) HSPC populations in the PB that were individually evaluable for the CXCR4 expression (Figure 3A). The CXCR4^+^ cell percentages in the HLA(−) HSPC population of the five patients (median, 8.8%; range, 2.1%–16.9%; mean fluorescence intensity [MFI] median, 211; MFI range, 19–333) were lower than those in their HLA(+) cell counterparts (median, 21.5%; range, 17.5%–27.7%; MFI median, 423; MFI range, 340‐708; Figure 3B,C). In contrast, B cells of the same patients highly expressed CXCR4, regardless of the HLA expression (Figure 3D,E). To determine whether or not the diminished CXCR4 expression on HSPCs was common to patients with escape clonal hematopoiesis, we studied PB HSPCs of five paroxysmal nocturnal hemoglobinuria (PNH) patients (PNH1–5) for whom both glycosylphosphatidylinositol (GPI)(−) and GPI(+) CD34^+^ cells were evaluable. The CXCR4^+^ cell percentages in GPI(−) HSPCs (median, 7.8%; range, 2.3%–12.5%; MFI median, 332; MFI range, 125–451) were significantly lower than in GPI(+) cells (median, 26.7%; range, 19.3%–32.5%; MFI median, 799; MFI range, 523–812) (Figure 4A,B). In contrast, B cells of the same PNH patients highly expressed CXCR4, regardless of the GPI expression (Figure 4C).

### 3.5 The CXCR4 expression on HSPCs of patients with MDS and AA patients with somatic mutations

**FIGURE 5:**
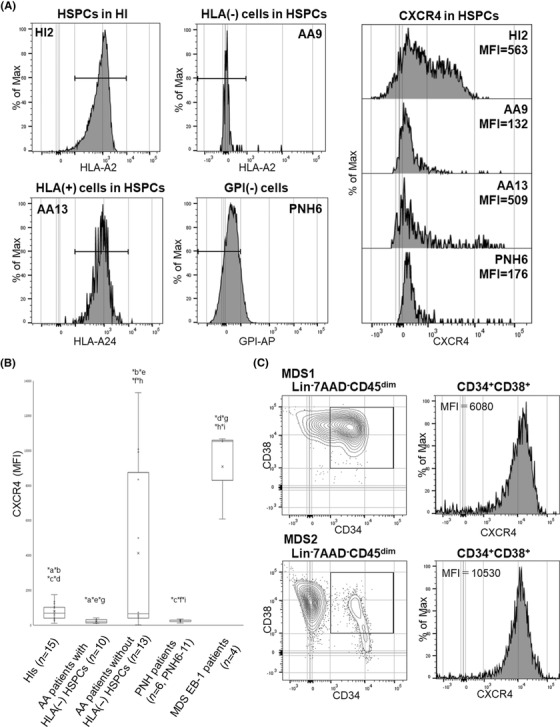
CXCR4 expression by hematopoietic stem progenitor cells (HSPCs) from aplastic anemia (AA) patients whose HSPCs consisted solely of HLA(−) cells, HLA(+) cells, or glycosylphosphatidylinositol (GPI)(−) cells, and CXCR4 expression by peripheral blood (PB) HSPCs from different patients. (A) CXCR4 expression on HSPCs. Each histogram shows the percentage of CXCR4^+^ cells in the total HSPCs in PB from a healthy individual (HI2), an AA patient with HLA(−) cells (AA9), an AA patient without HLA(−) cells (AA13), and a paroxysmal nocturnal hemoglobinuria (PNH) patient (PNH6). (B) The CXCR4 expression on whole HSPCs from patients with various disease states. ^*^ *p *< 0.05. (C) The CXCR4 expression on HSPCs of two patients with myelodysplastic syndromes (MDS) (MDS1 and MDS2). EB‐1, excess blast‐1; Lin, lineage; MFI, mean fluorescence intensity.^*^a–^*^i, denote that there is a significant difference between the two bar graphs

The diminished CXCR4 expression on HLA(−) and GPI(−) HSPCs contrasted with increased CXCR4 expression on HSPCs of patients with MDS previously reported [20, 21]. We therefore hypothesized that the intensity of CXCR4 expression on HSPCs might be useful for discriminating benign clonal hematopoiesis from a premalignant entity in patients with BM failure. To test this hypothesis, we screened PB HSPCs from patients with various types of BM failure, including MDS, for the CXCR4 expression. The percentage of CXCR4^+^ cells in HIs was 12.3%–49.9% (*n* = 15, median 43.2%; MFI median, 684; MFI range, 115–1758; Figure 5A,B). The median percentage of CXCR4^+^ cells in the whole PB HSPCs of ten patients (AA1‐10) with HLA(−) granulocytes was 8.8% (range, 2.1%–16.9%; MFI median, 192; MFI range, 108–416; Figure 5A,B). The percentage was significantly lower than that of AA patients without HLA(−) granulocytes (*n* = 13; median, 36.2%; range, 14.3%–88.7%; MFI median, 596; MFI range, 182–13,320; *p *< 1.0 × 10^−2^) or HIs (*p *< 1.0 × 10^−3^, Figure 5A,B). The percentage of CXCR4^+^ cells was also much lower among GPI(−) CD34^+^ cells of the six patients (PNH6–11) with hemolytic PNH than in HIs (median, 7.8%; range, 2.1%–8.7%; MFI median, 299; MFI range, 176–354; *p *< 1.0 × 10^−3^, Figure 5A,B).

Consistent with previous reports [20, 21], the percentages of CXCR4(+) HSPCs were increased in four patients with MDS (median, 66.5%; range, 54.2%–88.9%; MFI median, 8305; MFI range, 5147–10,675; Figure 5B,C) who had low percentage blasts (1.1%–2.1%) in PB.

**FIGURE 6:**
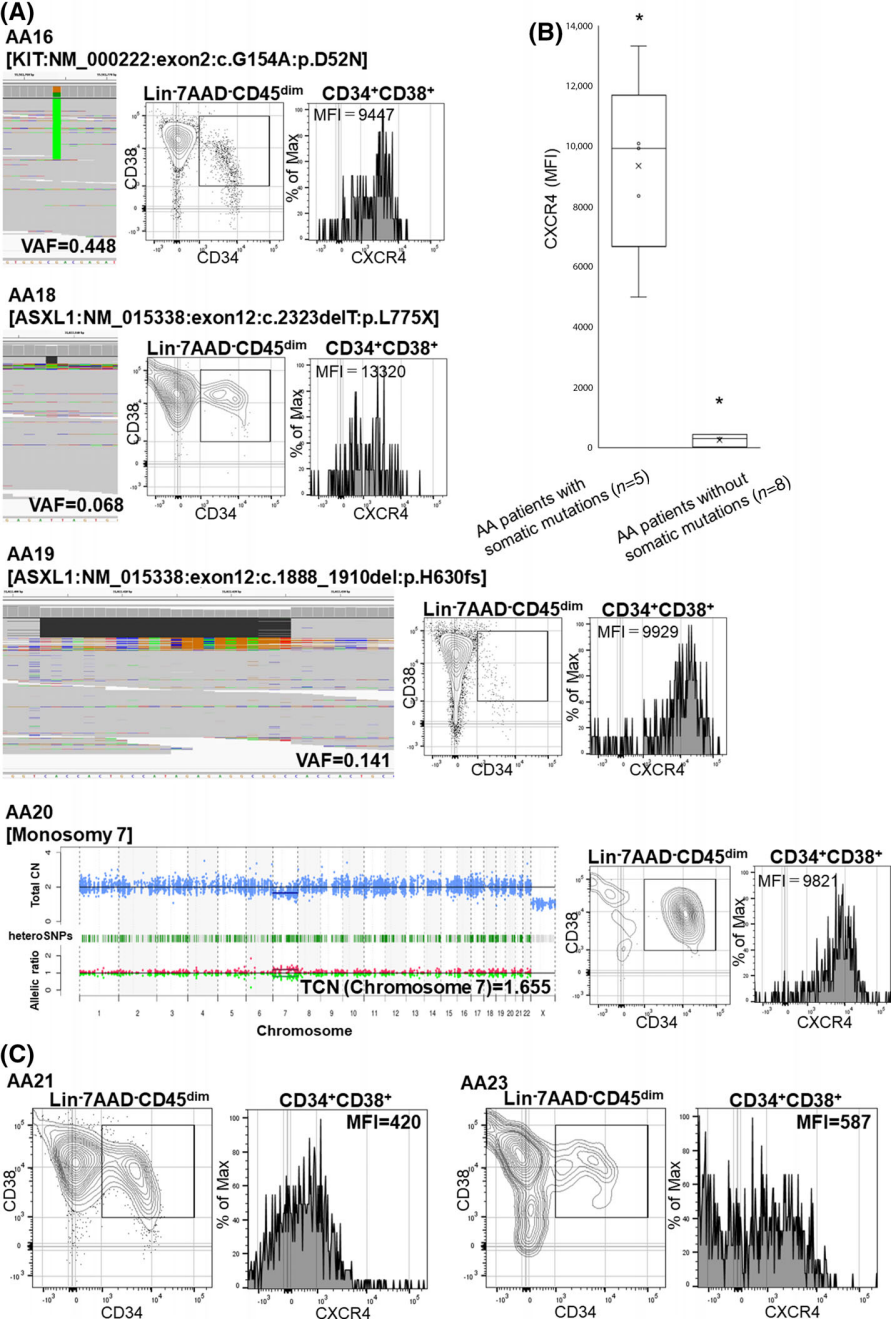
CXCR4 expression by CD34^+^CD38^+^ cells in peripheral blood (PB) from aplastic anemia (AA) patients with or without somatic mutations. (A) The results of targeted sequencing exhibiting mutated loci of *KIT* (AA16), *ASXL1* (AA18 and AA19), and monosomy 7 (AA20) are shown with flow cytometry (FCM) histograms. (B) The CXCR4 expression on hematopoietic stem progenitor cells (HSPCs) from five patients with somatic mutations and eight patients without somatic mutations. ^*^ *p *< 0.05. (C) CXCR4 expression levels by CD34^+^CD38^+^ cells from two AA patients without somatic mutations are shown (AA21 and AA23). HI, healthy individual; Lin, lineage; TCN, total copy number; VAF, variant allele frequency

Of the 13 AA patients without HLA(−) granulocytes, 5 (AA16–20) had clonal hematopoiesis due to somatic mutations in *c‐kit* (*n* = 1), *t*(8;21) (*n* = 1), *ASXL1* (*n* = 2), and monosomy 7 (*n* = 1), with variant allele frequencies of 0.448, 0.153, 0.141, 0.068, and 0.345 (AA16–20; Figure 6A). The percentages of CXCR4^+^ cells in PB CD34^+^ cells in these five patients (AA16–20) were significantly higher (61.4%, 78.5%, 88.7%, 73.9%, and 80.8%, respectively; MFI median, 10,009; MFI range, 9447–13,320) than in the remaining eight patients (AA11‐15, 21–23) without somatic mutations (median, 29.3%; range, 14.3%–37.3%; MFI median, 464; MFI range, 182–721; *p *< 1.0 × 10^−4^, Figure 6B,C).

## 4 DISCUSSION

Taking advantage of the phenotypic difference between HLA(−) CMPs and HLA(+) CMPs from AA patients whose granulocytes consisted of almost all HLA(−) cells, this study demonstrated for the first time that the diminished expression of CXCR4 was a feature of HSPCs that actively support hematopoiesis in humans. The CXCR4 expression level of HLA(+) CMPs that did not contribute to hematopoiesis of the same patients was comparable to that of HIs. This finding was substantiated by the decrease in the CXCR4 expression on the GPI(−) HSPCs of patients with florid PNH, which constituted clonal hematopoiesis with GPI(−) blood cells similarly to HLA(−) HSPCs. The normal expression of CXCR4 on the B cells of AA patients and mice engrafted with CXCR4^low^ iPSC‐HSPCs indicate that both HLA(−) and GPI(−) HSPCs epigenetically downregulated CXCR4. Given that the interaction of CXCR4^+^ HSPCs with *CXCL12* on BM stromal cells allows HSPCs to remain dormant, the epigenetic downregulation of CXCR4 may be a physiological phenomenon required for HSPCs to undergo differentiation into mature blood cells.

The percentages of HLA(−) cells in the CMPs of the three AA patients were much lower than those in the mature granulocytes of the same patients. This discrepancy in the percentage of affected cells between CD34^+^ cells and granulocytes was not seen in GPI(−) cells (data not shown). We recently demonstrated that HLA(−) HSPCs differed from GPI(−) HSPCs in hierarchy and in the sensitivity to immune attack in patients with AA, and the immune attack against HPCS persisted subclinically, even in patients who were in complete remission, allowing for the expansion of HLA(−) HSPCs [22]. The persistent immune pressure by cytotoxic T lymphocytes (CTLs) may eliminate the progeny of HLA(+) CMPs, leading to the predominance of HLA(−) cells in more committed progenitor cells than CMPs.

A previous study reported that CXCR4 on GPI(−) HSPCs was diminished due to the failure of lipid raft formation, and GPI(−) HSPCs might thereby be more likely to commit than GPI(+) HSPCs [23, 24, 25]. Although we confirmed that the CXCR4 expression on GPI(−) HSPCs of PNH patients was much lower than that on GPI(+) HSPCs of HIs, the failed lipid raft formation cannot explain the comparable expression levels of CXCR4 between GPI(−) B and GPI(+) B cells of the same patients. It is more reasonable to interpret that the decrease in the CXCR4 expression level, which was seen in both HLA(−) and GPI(−) HSPCs, is a common feature of HSPCs that actively support hematopoiesis in humans.

We observed the increased expression of CXCR4 by PB CD34^+^ cells not only in MDS patients but also in AA patients who exhibited clonal hematopoiesis associated with somatic mutations of myeloid malignancy‐related genes. CXCR4^high^ HSPCs have been shown to be more likely to migrate to the BM niche in comparison to CXCR4^low^ HSPCs, and their preferential binding to stromal cells expressing *CXCL12* promotes the proliferation of abnormal HSPCs via activation of the *PI3K/Akt* pathway [26, 27, 28]. Although the mechanism underlying the upregulation of CXCR4 in HSPCs with somatic mutations remains unclear, the increased CXCR4 expression on PB HSPCs may help identify abnormal HSPCs that may transform into MDS/acute myeloid leukemia [20, 26, 29, 30].

The results of the present study have significant clinical relevance. Although the hematopoiesis of patients with AA who are in remission is often clonal or oligoclonal, it is not easy to detect somatic mutations in leukocytes in clinical practice. Clonal hematopoiesis may be supported by HSPCs that are affected by some gene mutations which cannot be revealed by targeted sequencing. Evaluating the CXCR4 expression on PB HSPCs may help distinguish premalignant clonal hematopoiesis from benign clonal hematopoiesis in patients with BM failure.

## CONFLICT OF INTEREST

All authors declare no conflicts of interest in association with the present study.

## ETHICS STATEMENT

The authors consciously assure that for this article the following statements are fulfilled:

1. This material is the authors' own original work that has not been previously published elsewhere.

2. The paper is not currently being considered for publication elsewhere.

3. The paper reflects the authors' own research and analysis in a truthful and complete manner.

4. The paper properly credits the meaningful contributions of coauthors.

5. The results are appropriately placed in the context of prior and existing research.

6. All sources used are properly disclosed. Literally copying of text must be indicated as such by using quotation marks and giving proper reference.

7. All authors have been personally and actively involved in substantial work leading to the paper and will take public responsibility for its content.

## AUTHOR CONTRIBUTION

Takamasa Katagiri and Shinji Nakao developed the concept of the study and designed the experiments. Takamasa Katagiri, Jorge Luis. Espinoza, Mizuho Uemori, Honoka Ikeda, Kohei Hosokawa, Ken Ishiyama, Takeshi Yoroidaka, Tatsuya Imi, Hiroyuki Takamatsu, Tatsuhiko Ozawa, Hiroyuki Kishi, Yasuhiko Yamamoto, Mahmoud Ibrahim. Elbadry, Yasuhito Nannya, and Seishi Ogawa performed the experiments and analyzed the data. Yoshinori Yoshida and Kazuhisa Chonabayashi established iPS cells. Katsuto Takenaka and Koichi Akashi established BRGS mice. Takamasa Katagiri and Shinji Nakao wrote the paper. All authors approved the final version of this paper.

## Supporting information

Supporting Information

## Data Availability

All data generated or analyzed during this study are included in this article and Supporting Information section. The data described in this article are openly available.
