# Supplementary material for: Hematopoietic stem progenitor cells with malignancy‐related gene mutations in patients with acquired aplastic anemia are characterized by the increased expression of CXCR4
Source: EJHaem. 2022 Jul 3;3(3):669–80. doi: 10.1002/jha2.515 (PMC9422028; doi:10.1002/jha2.515)
Supplement: Supplementary file 1 — Supporting Information [file JHA2-3-669-s001.docx]

**Supplemental information**

**Hematopoietic stem progenitor cells with malignancy-related gene mutations in patients with acquired aplastic anemia are characterized by the increased expression of CXCR4**

Takamasa Katagiri^1)^, J Luis Espinoza^2)^, Mizuho Uemori^1)^, Honoka Ikeda^1)^, Kohei Hosokawa^3)^, Ken Ishiyama^3)^, Takeshi Yoroidaka^3)^, Tatsuya Imi^3)^, Hiroyuki Takamatsu^3)^, Tatsuhiko Ozawa^4)^, Hiroyuki Kishi^4)^, Yasuhiko Yamamoto^5)^, Mahmoud I. Elbadry^6)^, Yoshinori Yoshida^7)^, Kazuhisa Chonabayashi^7)8)^, Katsuto Takenaka^9)^, Koichi Akashi^10)^, Yasuhito Nannya^11)12)^, Seishi Ogawa^12)13)14)^ and Shinji Nakao^3)++^

^1)^ Department of Clinical Laboratory Science, Graduate School of Medical Science, Institute of Medical Pharmaceutical and Health Sciences, Kanazawa University, 5-11-80 Kodatsuno, Kanazawa, Ishikawa 920-0942 Japan.

^2)^ Department of Occupational Therapy, Graduate School of Medical Science, Institute of Medical Pharmaceutical and Health Sciences, Kanazawa University, 5-11-80 Kodatsuno, Kanazawa, Ishikawa 920-0942 Japan.

^3)^ Department of Hematology, Faculty of Medicine, Institute of Medical Pharmaceutical and Health Sciences, Kanazawa University, 13-1 Takara-machi, Kanazawa, Ishikawa 920-8641, Japan.

^4)^ Department of Immunology, Faculty of Medicine, Academic Assembly, University of Toyama, 2630 Sugitani, Toyama-city, Toyama 930-0194, Japan.

^5)^ Department of Biochemistry and Molecular Vascular Biology, Kanazawa University Graduate School of Medical Sciences, 13-1 Takara-machi, Kanazawa, Ishikawa 920-8641, Japan.

^6)^ Department of Internal Medicine, Division of Hematology, Faculty of Medicine, Sohag University, Sohag University Street Nasser City, 82524 Egypt.

^7)^ Center for iPS Cell Research and Application, Kyoto University, 53 Kawahara-cho, Shogoin, Sakyo-ku, Kyoto 606-8507, Japan.

^8)^ Department of Hematology and Oncology, Graduate School of Medicine, Kyoto University,

54 Kawahara-cho, Shogoin, Sakyo-ku, Kyoto 606-8507, Japan.

^9)^ Department of Hematology, Clinical Immunology and Infectious Diseases, Ehime University Graduate School of Medicine, Shitsukawa, Toon, Ehime 791-0295 Japan.

^10)^ Medicine and Biosystemic Science, Kyushu University Graduate School, 3-1-1 Maidashi, Higashi-ku, Fukuoka-city, Fukuoka 812-8582 Japan.

^11)^ Division of Hematopoietic Disease Control, Institute of Medical Science, University of Tokyo, 4-6-1 Shiroganedai Minato-ku, Tokyo 108-8639, Japan.

^12)^ Department of Pathology and Tumor Biology, Kyoto University, Yoshida-Konoe-cho, Sakyo-ku, Kyoto 606-8501, Japan.

^13)^ Institute for the Advanced Study of Human Biology (WPI-ASHBi), Kyoto University, Yoshida-Konoe-cho, Sakyo-ku, Kyoto 606-8501, Japan.

^14)^ Department of Medicine, Centre for Hematology and Regenerative Medicine, Karolinska Institute, Stockholm 171 77, Sweden.

**RUNNING HEADS**: Increased CXCR4 expression on HSPCs with mutations

**++CORRESPONDING AUTHOR**

Shinji Nakao, M.D., Ph.D.

Department of Hematology, Faculty of Medicine, Institute of Medical Pharmaceutical and Health Sciences, Kanazawa University, Takara-machi 13-1, Kanazawa, Ishikawa 920-8641, Japan

Phone: +81 762652274,

Fax: +81 762344252

E-mail: snakao8205@staff.kanazawa-u.ac.jp

**MATERIALS AND METHODS**

Patients

Flow cytometry analysis and cell sorting

Microarray analysis

**SUPPLEMENTAL TABLES**

Table S1. Characteristics of patients and healthy individuals

Table S2. Monoclonal antibodies used for flow cytometry.

Table S3. Genes differentially expressed by HLA(-) and HLA(+) CMPs.

**SUPPLEMENTAL FIGURES**

Figure S1. A work flow to determine the gene expression profile by CMPs from AA patients with microarray analysis.

Figure S2. The percentage of HLA(-) cells in CD34^+^ cells of BM and granulocytes from AA patients.

Figure S3. The percentage of HLA(-) cells in CD34^+^ subsets of BM and granulocytes in PB from three AA patients.

**REFERENCES**

**MATERIALS AND METHODS**

**Patients**

BM and PB samples were obtained from a total of 38 patients with hematologic diseases including AA (n=23), paroxysmal nocturnal hemoglobinuria (PNH) (n=11) and MDS (n=4) during a period from 2018 to 2021. All samples were obtained from AA patients in remission after treatment. **Supplemental Table 1** shows the characteristics of patient and healthy individuals. Severe AA was diagnosed when at least 2 of the following criteria were met: neutrophil count <0.5 × 10^9^ /L, platelet count < 20 × 10^9^ /L and reticulocyte count < 20 × 10^9^ /L,^1^ and that very severe AA was defined as a neutrophil count < 0.2 × 10^9^/L in addition to the criteria for severe AA.^2^ Responses to immunosuppressive therapy were evaluated according to established criteria.^3^ Genotyping of *HLA-A*, *HLA-B*, *HLA-C*, and *HLA-DRB1* alleles were performed using the polymerase chain reaction (PCR) sequence-specific oligonucleotide method, as previously described.^4^ MDS was diagnosed based on the 2016 World Health Organization (WHO) classification.^5,6^ We defined individuals as healthy controls whose were older than 20 years and were free of any underlying medical conditions. PB (n=15) and BM (n=11) samples from 26 healthy volunteers were used to assess the CXCR4 expression on their HSPCs. The male to female ratios were 1:1 and 1:1.2, and the median ages were 55.5 and 56 years, ranging from 24 to 85 and 26 to 79 years in AA patients and healthy individuals, respectively.

All patients and healthy volunteers provided their informed consent in accordance with the Declaration of Helsinki. This study was approved by the ethics committee of Kanazawa University Institute of Medical, Pharmaceutical, and Health Sciences (No. 2016-284 and No.2018-017).

**Flow cytometry analysis and cell sorting**

HLA allelic expression by different subsets of cells, including CD34^+^ cell subsets in BM, CD34^+^ CD38^+^ cells in PB, and PB granulocytes were assessed using flow cytometry (FCM) with anti-HLA-A allele-specific mAbs, A2 (FH0037; One Lambda, Inc., CA, USA), A24 (FH0964; One Lambda), A26 (BIH0048; One Lambda, Inc., CA, USA), or A31 (BIH0087; One Lambda). CD34^+^ cell subsets in BM and CD34^+^ HSPCs in PB were defined as follows;, common myeloid progenitors (CMPs) as CD34^+^CD38^+^CD135^+^CD45RA^-^, megakaryocyte-erythroid progenitors (MEPs) as CD34^+^CD38^+^CD135^-^CD45RA^-^, granulocytic macrophage progenitors (GMPs) as CD34^+^CD38^+^CD135^+^CD45RA^+^,^7^ CD34^+^ HSPCs in PB as lineage^-^CD45^dim^CD34^+^CD38^+^.

The CXCR4 expression levels on different cell populations, including CD34^+^ cell subpopulations in BM of patients harboring HLA(-) cells, CD34^+^ HSPCs in PB, mature cells in PB and HSPCs induced from induced pluripotent stem cells (iPSCs), were determined using a monoclonal antibody specific to human CD184 (562448; Becton Dickinson, Franklin Lakes, NJ, USA) in combination with other antibodies. Monoclonal antibodies used for this study are provided in **Supplemental Table 2**. FCM was performed using a FACS Canto II® instrument (Becton Dickinson, Franklin Lakes, NJ, USA) and data was analyzed using the FlowJo 10.7.1 software program (Tree Star, Inc., Ashland, OR, USA), as previously described before.^8^ Paired fractions of BM, including HLA(-) CMPs and CMPs that retained the HLA-A allele (HLA[+] CMPs) were sorted using a FACSAria™ Fusion instrument (BD Biosciences, Franklin Lakes, NJ, USA). The sorted cells were subjected to RNA extraction using an RNeasy Micro Kit (Qiagen, Hilden, Germany) (**Supplemental Figure 1**).

**Microarray analysis**

As we used the small number of patients for the analyses, Bonferroni correction was used for multiplicity adjusting and significance analysis of microarrays (SAM), that assigns a score to each gene on the basis of change in gene expression relative to the standard deviation of the measurements according to the manufacturer setting. For genes with scores greater than an adjustable threshold, SAM uses permutations of the measurements to estimate the percentage of genes identified by chance, the false discovery rate, as previously described.^9^

**SUPPLEMENTAL TABLES**

**Table S1. Characteristics of patients and healthy individuals**

|  | Age | Follow-up period (years) | Gender | Dx | Severity | | Classification | Treatment | Response | RBC (10^12^/L) | Hb (g/dL) | WBC (10^9^/L) | Plt (10^9^/L) | HLA(-) in Gs (%) | GPI(-) in Gs (%) | Chromosomal abnormalities |
| --- | --- | --- | --- | --- | --- | --- | --- | --- | --- | --- | --- | --- | --- | --- | --- | --- |
| AA1 | 64 | 15 | M | AA | SAA | | Idiopathic | ATG, CsA | CR | 4.53 | 14.9 | 5.29 | 216 | 94.4 | － | 45,X,-Y 12/20; 46,XY,t(1;10)(q21;q24) 2/20 |
| AA2 | 33 | 19 | M | AA | SAA | | Idiopathic | ATG, CsA | CR | 4.65 | 15.1 | 2.72 | 158 | 60.3 | 8.1 | Normal karyotype |
| AA3 | 56 | 29 | M | AA | NSAA | | Idiopathic | CsA, AS | PR | 3.98 | 14.1 | 5.53 | 106 | 88.4 | － | Normal karyotype |
| AA4 | 60 | 7 | M | AA | NSAA | | Idiopathic | CsA | CR | 3.03 | 10.6 | 4.98 | 166 | 91.2 | 3.6 | Normal karyotype |
| AA5 | 80 | 30 | F | AA | NSAA | | Idiopathic | CsA | CR | 4.22 | 13.3 | 4.89 | 122 | 12.3 | － | Normal karyotype |
| AA6 | 51 | 5 | F | AA | SAA | | Idiopathic | ATG, CsA | CR | 3.47 | 11.6 | 3.65 | 117 | 26.8 | － | Normal karyotype |
| AA7 | 50 | 15 | M | AA | SAA | | Idiopathic | AS | CR | 3.72 | 12.6 | 4.16 | 183 | 54.2 | － | Normal karyotype |
| AA8 | 67 | 6 | F | AA | SAA | | Idiopathic | ATG, CsA | PR | 4.13 | 13.3 | 4.66 | 112 | 32.2 | － | Normal karyotype |
| AA9 | 44 | 19 | F | AA | NSAA | | Idiopathic | CsA | CR | 4.09 | 10 | 3.33 | 145 | 98.1 | － | Normal karyotype |
| AA10 | 85 | 17 | F | AA | NSAA | | Idiopathic | CsA | CR | 3.68 | 12.3 | 4.99 | 109 | 99.1 | － | Normal karyotype |
| AA11 | 31 | 15 | F | AA | NSAA | | Idiopathic | CsA | CR | 3.13 | 11.1 | 2.45 | 274 | － | － | Normal karyotype |
| AA12 | 67 | 17 | F | AA | NSAA | | Idiopathic | CsA, EPAG | NR | 3.34 | 11.6 | 4.17 | 90 | － | － | Normal karyotype |
| AA13 | 65 | 24 | F | AA | NSAA | | Idiopathic | EPAG, ROMI | PR | 4.02 | 14.2 | 3.87 | 39 | － | － | Normal karyotype |
| AA14 | 24 | 3 | M | AA | SAA | | Idiopathic | ATG, CsA, EPAG | CR | 4.94 | 14.6 | 5.4 | 274 | － | － | Normal karyotype |
| AA15 | 47 | 3 | M | AA | NSAA | | Idiopathic | EPAG, ROMI | PR | 4.11 | 14.1 | 5.79 | 148 | － | － | Normal karyotype |
| AA16 | 49 | 13 | F | AA | NSAA | | Idiopathic | CsA, EPAG, ROMI | PR | 3.27 | 11.9 | 2.81 | 28 | － | － | Normal karyotype |
| AA17 | 29 | 25 | M | AA | NSAA | | Idiopathic | AS | PR | 2.98 | 12.5 | 1.87 | 125 | － | － | Normal karyotype |
| AA18 | 52 | 6 | F | AA | NSAA | | Idiopathic | CsA, AS, ROMI | PR | 3.1 | 10.7 | 3.77 | 41 | － | － | Normal karyotype |
| AA19 | 55 | 3 | M | AA | SAA | | Idiopathic | ATG, CsA, EPAG | CR | 5.17 | 16.4 | 3.33 | 93 | － | － | Normal karyotype |
| AA20 | 57 | 5 | M | AA | NSAA | | Idiopathic | CsA, | PR | 3.21 | 10.9 | 3.65 | 101 | － | － | 46,XY,-7 13/20 |
| AA21 | 65 | 25 | F | AA | NSAA | | Idiopathic | CsA, EPAG, ROMI | NR | 3.59 | 12.2 | 4.7 | 6 | － | － | Normal karyotype |
| AA22 | 51 | 12 | F | AA | NSAA | | Idiopathic | ATG, CsA | PR | 4.28 | 13.9 | 5.97 | 196 | － | － | Normal karyotype |
| AA23 | 55 | 28 | F | AA | NSAA | | Idiopathic | ATG, CsA, AS | PR | 3.83 | 12.1 | 2.85 | 103 | － | － | Normal karyotype |
| PNH1 | 27 | 21 | M | PNH | Moderate | | AA/PNH | CsA, Ravulizumab | PR | 1.77 | 7 | 3.55 | 134 | － | 21.2 | Normal karyotype |
| PNH2 | 48 | 2 | F | PNH | Moderate | | AA/PNH | No treatment | NA | 4.25 | 14.1 | 5.96 | 118 | － | 38.8 | Normal karyotype |
| PNH3 | 72 | 2 | M | PNH | Moderate | | AA/PNH | CsA, ROMI, Ravulizumab | PR | 2.53 | 9.9 | 1.9 | 31 | － | 50.1 | Normal karyotype |
| PNH4 | 62 | 13 | M | PNH | Moderate | | AA/PNH | Ravulizumab | PR | 2.61 | 9.2 | 4.26 | 6 | － | 57.1 | Normal karyotype |
| PNH5 | 73 | 38 | F | PNH | | Moderate | AA/PNH | Ravulizumab | NE | 2.33 | 7.5 | 2.16 | 75 | － | 16.8 | Normal karyotype |
| PNH6 | 43 | 6 | F | PNH | | Moderate | AA/PNH | Ravulizumab | NE | 2.76 | 10.7 | 3 | 96 | － | 85.4 | Normal karyotype |
| PNH7 | 70 | 31 | M | PNH | | Moderate | AA/PNH | Ravulizumab | NE | 1.72 | 6.6 | 2.6 | 117 | － | 81.4 | 46,XY,del(20)(q11.2q13.3) 17/20 |
| PNH8 | 33 | 4 | M | PNH | | Moderate | Classical PNH | Ravulizumab | NE | 3.47 | 12 | 4.6 | 120 | NA | 96.3 | Normal karyotype |
| PNH9 | 66 | 30 | F | PNH | | Moderate | AA/PNH | Ravulizumab | NE | 3 | 9.9 | 3.99 | 152 | － | 99.3 | Normal karyotype |
| PNH10 | 51 | 13 | M | PNH | | Moderate | AA/PNH | CsA, Eculizumab | PR | 2.93 | 11.2 | 3.35 | 169 | － | 88.8 | Normal karyotype |
| PNH11 | 57 | 32 | M | PNH | | Moderate | AA/PNH | Ravulizumab | NE | 2.68 | 9.5 | 3.38 | 211 | － | 98.2 | Normal karyotype |
| MDS1 | 70 | 1 | F | MDS | | NA | MDS-EB-1 | Aza | NE | 3.43 | 11 | 3.8 | 16 | NA | NA | 47, XX, +8 3/20 |
| MDS2 | 66 | 3 | F | MDS | | NA | MDS-EB-1 | No treatment | NE | 3.34 | 11.3 | 2.72 | 167 | NA | NA | 46,XX,del(20)(q11.2q13.3) 15/20 |
| MDS3 | 53 | 11 | M | MDS | | NA | MDS-EB-1 | No treatment | NE | 3.95 | 11.2 | 3.26 | 34 | NA | NA | 47,XY,+8 2/20 |
| MDS4 | 59 | 11 | M | MDS | | NA | MDS-EB-1 | No treatment | NE | 3.08 | 11.9 | 2.47 | 2 | NA | NA | 47,XY,+8 9/20 |
| HI1 | 43 | NA | M | NA | | NA | NA | NA | NA | NA | NA | NA | NA | NA | NA |  |
| HI2 | 44 | NA | F | NA | | NA | NA | NA | NA | NA | NA | NA | NA | NA | NA |  |
| HI3 | 38 | NA | M | NA | | NA | NA | NA | NA | NA | NA | NA | NA | NA | NA |  |
| HI4 | 64 | NA | F | NA | | NA | NA | NA | NA | NA | NA | NA | NA | NA | NA |  |
| HI5 | 60 | NA | F | NA | | NA | NA | NA | NA | NA | NA | NA | NA | NA | NA |  |
| HI6 | 33 | NA | F | NA | | NA | NA | NA | NA | NA | NA | NA | NA | NA | NA |  |
| HI7 | 75 | NA | M | NA | | NA | NA | NA | NA | NA | NA | NA | NA | NA | NA |  |
| HI8 | 68 | NA | M | NA | | NA | NA | NA | NA | NA | NA | NA | NA | NA | NA |  |
| HI9 | 67 | NA | M | NA | | NA | NA | NA | NA | NA | NA | NA | NA | NA | NA |  |
| HI10 | 64 | NA | F | NA | | NA | NA | NA | NA | NA | NA | NA | NA | NA | NA |  |
| HI11 | 64 | NA | F | NA | | NA | NA | NA | NA | NA | NA | NA | NA | NA | NA |  |
| HI12 | 52 | NA | F | NA | | NA | NA | NA | NA | NA | NA | NA | NA | NA | NA |  |
| HI13 | 79 | NA | M | NA | | NA | NA | NA | NA | NA | NA | NA | NA | NA | NA |  |
| HI14 | 76 | NA | F | NA | | NA | NA | NA | NA | NA | NA | NA | NA | NA | NA |  |
| HI15 | 58 | NA | M | NA | | NA | NA | NA | NA | NA | NA | NA | NA | NA | NA |  |
| HI16 | 42 | NA | F | NA | | NA | NA | NA | NA | NA | NA | NA | NA | NA | NA |  |
| HI17 | 55 | NA | M | NA | | NA | NA | NA | NA | NA | NA | NA | NA | NA | NA |  |
| HI18 | 38 | NA | F | NA | | NA | NA | NA | NA | NA | NA | NA | NA | NA | NA |  |
| HI19 | 60 | NA | M | NA | | NA | NA | NA | NA | NA | NA | NA | NA | NA | NA |  |
| HI20 | 57 | NA | F | NA | | NA | NA | NA | NA | NA | NA | NA | NA | NA | NA |  |
| HI21 | 48 | NA | F | NA | | NA | NA | NA | NA | NA | NA | NA | NA | NA | NA |  |
| HI22 | 36 | NA | M | NA | | NA | NA | NA | NA | NA | NA | NA | NA | NA | NA |  |
| HI23 | 58 | NA | F | NA | | NA | NA | NA | NA | NA | NA | NA | NA | NA | NA |  |
| HI24 | 54 | NA | F | NA | | NA | NA | NA | NA | NA | NA | NA | NA | NA | NA |  |
| HI25 | 28 | NA | M | NA | | NA | NA | NA | NA | NA | NA | NA | NA | NA | NA |  |
| HI26 | 26 | NA | M | NA | | NA | NA | NA | NA | NA | NA | NA | NA | NA | NA |  |
| Abbreviations: AA, aplastic anemia; PNH, paroxysmal nocturnal hematuria; MDS, myelodysplastic syndromes; HI, healthy individual; M, male; F, female; Dx, diagnosis; SAA, severe aplastic anemia; NSAA, non-severe aplastic anemia; EB-1, excess blast-1; ATG, antithymocyte globulin; CsA, cyclosporine; AS, anabolic steroids; EPAG, eltrombopag; ROMI, Romiplostim; Aza, azacytidine; Response, Response to treatment; CR, complete remission; PR, partial remission; NR, no response: NE, not evaluable; NA, not applicable; RBC, red blood cells; Hb, hemoglobin; WBC, white blood cell; Plt, platelet; HLA(-), HLA-A allele deficient; GPI(-), glycosylphosphatidylinositol-anchored proteins deficient; Gs, granulocytes; -, not detected. | | | | | | | | | | | | | | | | |
|  |  |  |  |  |  |  |  |  |  |  |  |  |  |  |  |  |
|  |  |  |  |  |  |  |  |  |  |  |  |  |  |  |  |  |

| Antigen | Isotype | Conjugate | Source |
| --- | --- | --- | --- |
| CD11b | IgG1 | PE | BD Biosciences |
| CD11b | IgG1 | APC | Beckman Coulter |
| CD135 | Mouse BALB/c IgG1 | PerCP-Cy™5.5 | BD Pharmingen |
| CD135 | Mouse BALB/c IgG1 | Alexa Fluor® 647 | BD Pharmingen |
| CD184 | Mouse BALB/c IgG2a | BV421 | BD Horizon |
| CD33 | Mouse IgG1 | APC | Beckman Coulter |
| CD34 | Mouse IgG1 | FITC | BD Pharmingen |
| CD34 | Mouse IgG1 | PE | BD Pharmingen |
| CD34 | Mouse IgG1 | APC-Cy™7 | BD Pharmingen |
| CD38 | Mouse IgG1 | PE | BD Pharmingen |
| CD38 | Mouse IgG2 | PE-Cy™7 | BD Pharmingen |
| CD38 | Mouse IgG1 | APC | BD Pharmingen |
| CD45 | Mouse IgG1 | V500 | BD Biosciences |
| CD45RA | Mouse IgG1 | PE-Cy™7 | BD Pharmingen |
| CD45RA | Mouse IgG2b | APC | BD Pharmingen |
| HLA-A2/28 | Mouse IgG2a | FITC | One Lambda |
| HLA-A25/26 | IgM | Biotin | ONE LAMBDA |
| HLA-A30/31 | Mouse IgM | Biotin | One Lambda |
| HLA-A9/24 | Mouse IgG2b | FITC | One Lambda |
| Human Lineage Cocktail 4 | Mouse IgG1 | FITC | BD Pharmingen |
| 7AAD | - | - | BD Pharmingen |
| SSEA-4 | Mouse IgG3 | PE | Biolegend |
| SSEA-4 | Mouse IgG3 | PE-Cy7 | Biolegend |
| Streptavidin | NA | PE | BD Biosciences |
| Abbreviations: Ig, immunoglobulin; PerCP-Cy5.5, peridinin-chlorophyll proteins-Cy5.5 tandem; PE, phycoerythrin; APC, allophycocyanin; APC-Cy7, allophycocyanin-Cy7 tandem; FITC, fluorescein isothiocyanate; PE-Cy7, phycoerythrin-Ct7 tandem; V500, Violet 500; BV421, BD Horizon Brilliant Violet 421. | | | |
|  |  |  |  |

**Table S2. Monoclonal antibodies used for flow cytometry**

**Table S3. Genes differentially expressed by HLA(-) and HLA(+) CMPs**

| **ProbeName** | **GeneSymbol** | **Regulation (CMPretain/CMPloss)** | **Cytoband** | **Entrez GeneID** | **Genbank Accession** | **Genomic Coordinates** | **TIGRID** | **UniGeneID** |
| --- | --- | --- | --- | --- | --- | --- | --- | --- |
|  |  |  |  |  |  |  |  |  |
| A_33_P3298024 | ABCC3 | down | hs\|17q21.33 | 8714 | NM_001144070 | chr17:48745220-48745279 | NP393613 | Hs.463421 |
| A_24_P140405 | ADAMTS3 | up | hs\|4q13.3 | 9508 | NM_014243 | chr4:73147061-73147002 | THC2475639 | Hs.590919 |
| A_33_P3392092 | ADCYAP1 | up | hs\|18p11.32 | 116 | NM_001099733 | chr18:911932-911991 | THC2655523 | Hs.531719 |
| A_23_P72387 | AFAP1 | down | hs\|4p16.1 | 60312 | NM_001134647 | chr4:7760626-7760567 | THC2491273 | Hs.529369 |
| A_33_P3238993 | AGFG1 | down | hs\|2q36.3 | 3267 | NM_001135187 | chr2:228419195-228419254 | THC2634102 | Hs.352962 |
| A_23_P205959 | ALDH1A3 | down | hs\|15q26.3 | 220 | NM_000693 | chr15:101456503-101456562 | NP1072657 | Hs.459538 |
| A_23_P166686 | AMOTL2 | down | hs\|3q22.2 | 51421 | NM_016201 | chr3:134074801-134074742 | THC2469043 | Hs.426312 |
| A_33_P3295358 | ANGPTL4 | down | hs\|19p13.2 | 51129 | NM_139314 | chr19:8439197-8439256 | THC2466722 | Hs.9613 |
| A_33_P3223592 | APOE | down | hs\|19q13.32 | 348 | NM_000041 | chr19:45412590-45412649 | THC2465276 | Hs.654439 |
| A_33_P3405023 | BLACE | down | hs\|7q36.3 | 338436 | AY166699 | chr7:155149777-155149718 | THC2570675 | Hs.521326 |
| A_23_P161439 | C10orf116 | down | hs\|10q23.2 | 10974 | NM_006829 | chr10:88730308-88730367 | THC2462530 | Hs.642660 |
| A_33_P3383236 | C2orf62 | down | hs\|2q35 | 375307 | NM_198559 | chr2:219232181-219232240 | THC2515065 | Hs.645453 |
| A_23_P63379 | CA14 | up | hs\|1q21.2 | 23632 | NM_012113 | chr1:150237369-150237428 | THC2471240 | Hs.528988 |
| A_24_P921366 | CALD1 | down | hs\|7q33 | 800 | NM_033138 | chr7:134655197-134655256 | NP1159719 | Hs.490203 |
| A_23_P218369 | CCL14 | down | hs\|17q12 | 6358 | NM_032963 | chr17:34310865-34310806 | THC2488869 | Hs.569800 |
| A_23_P55270 | CCL18 | down | hs\|17q12 | 6362 | NM_002988 | chr17:34398680-34398739 | THC2464854 | Hs.143961 |
| A_23_P89431 | CCL2 | down | hs\|17q12 | 6347 | NM_002982 | chr17:32584050-32584109 | THC2489756 | Hs.303649 |
| A_23_P207456 | CCL8 | down | hs\|17q12 | 6355 | NM_005623 | chr17:32648102-32648161 | THC2474860 | Hs.271387 |
| A_23_P402670 | CD1A | down | hs\|1q23.1 | 909 | NM_001763 | chr1:158227237-158227296 | THC2471110 | Hs.1309 |
| A_24_P305345 | CD209 | down | hs\|19p13.2 | 30835 | NM_021155 | chr19:7805407-7805348 | THC2537914 | Hs.278694 |
| A_33_P3224250 | CLCC1 | up | hs\|1p13.3 | 23155 | NM_001048210 | chr1:109472215-109472156 |  | Hs.658489 |
| A_24_P235988 | CLEC7A | down | hs\|12p13.2 | 64581 | NM_197947 | chr12:10269408-10269366 | THC2485854 | Hs.143929 |
| A_33_P3256778 | CNTF | down | hs\|11q12.1 | 1270 | NM_000614 | chr11:58393143-58393202 | THC2478967 | Hs.524920 |
| A_23_P216361 | COL14A1 | down | hs\|8q24.12 | 7373 | NM_021110 | chr8:121383648-121383707 | THC2670146 | Hs.409662 |
| A_33_P3304668 | COL1A1 | down | hs\|17q21.33 | 1277 | NM_000088 | chr17:48261568-48261509 | THC2549101 | Hs.172928 |
| A_24_P277934 | COL1A2 | down | hs\|7q21.3 | 1278 | NM_000089 | chr7:94058643-94058702 | THC2564543 | Hs.489142 |
| A_23_P19663 | CTGF | down | hs\|6q23.2 | 1490 | NM_001901 | chr6:132270189-132270130 | NP095144 | Hs.410037 |
| A_23_P18452 | CXCL9 | down | hs\|4q21.1 | 4283 | NM_002416 | chr4:76922855-76922796 | THC2461130 | Hs.77367 |
| A_24_P331150 | CYP4F22 | up | hs\|19p13.12 | 126410 | NM_173483 | chr19:15662720-15662779 | THC2479532 | Hs.156452 |
| A_23_P64873 | DCN | down | hs\|12q21.33 | 1634 | NM_001920 | chr12:91539893-91539834 | THC2522476 | Hs.706674 |
| A_23_P4494 | DSC2 | down | hs\|18q12.1 | 1824 | NM_024422 | chr18:28651609-28650796 | THC2462770 | Hs.95612 |
| A_33_P3243028 | DSPP | down | hs\|4q22.1 | 1834 | NM_014208 | chr4:88537422-88537481 | NP288484 | Hs.678914 |
| A_23_P119478 | EBI3 | down | hs\|19p13.3 | 10148 | NM_005755 | chr19:4237260-4237319 | THC2737205 | Hs.501452 |
| A_23_P154806 | EPB41L1 | down | hs\|20q11.23 | 2036 | NM_012156 | chr20:34818126-34818185 | THC2523234 | Hs.437422 |
| A_23_P136347 | EPS8 | down | hs\|12p12.3 | 2059 | NM_004447 | chr12:15774235-15774176 | THC2468128 | Hs.591160 |
| A_33_P3222501 | FAM157A | down | hs\|3q29 | 728262 | NM_001145248 | chr3:197896683-197896742 |  | Hs.730011 |
| A_32_P108254 | FAM20A | down | hs\|17q24.2 | 54757 | NM_017565 | chr17:66533427-66533368 | THC2476784 | Hs.268874 |
| A_33_P3234678 | FAM74A3 | down | hs\|9p13.1 | 728495 | NR_026801 | chr9:40722511-40722570 | THC2488644 | Hs.632659 |
| A_23_P211631 | FBLN1 | down | hs\|22q13.31 | 2192 | NM_006486 | chr22:45996933-45996992 | THC2575489 | Hs.24601 |
| A_33_P3710442 | FLJ11710 | up | hs\|17q22 | 79904 | AK021772 | chr17:55357314-55357373 | THC2653222 | Hs.657294 |
| A_23_P47709 | FOLR2 | down | hs\|11q13.4 | 2350 | NM_000803 | chr11:71932884-71932943 | THC2462702 | Hs.433159 |
| A_24_P32935 | FOLR2 | down | hs\|11q13.4 | 2350 | NM_000803 | chr11:71929635-71929694 | THC2783610 | Hs.433159 |
| A_33_P3247042 | FPR3 | down | hs\|19q13.41 | 2359 | NM_002030 | chr19:52329200-52329259 | THC2637585 | Hs.445466 |
| A_23_P363778 | FRZB | down | hs\|2q32.1 | 2487 | NM_001463 | chr2:183699679-183699620 | THC2494250 | Hs.128453 |
| A_23_P146922 | GAS6 | down | hs\|13q34 | 2621 | NM_000820 | chr13:114531609-114531550 | THC2506281 | Hs.646346 |
| A_23_P84666 | GDPD1 | up | hs\|17q22 | 284161 | NM_182569 | chr17:57335133-57335192 | NP495966 | Hs.631744 |
| A_33_P3317580 | GFRA2 | down |  | 2675 | NM_001495 | chr8_gl000197_random:24211-24270 | THC2471745 | Hs.441202 |
| A_33_P3255304 | GGT5 | down | hs\|22q11.23 | 2687 | NM_001099781 | chr22:24615682-24615623 | THC2605924 | Hs.437156 |
| A_23_P134426 | GPNMB | down | hs\|7p15.3 | 10457 | NM_001005340 | chr7:23314496-23314555 | THC2476654 | Hs.190495 |
| A_23_P324327 | GPRC5B | down | hs\|16p12.3 | 51704 | NM_016235 | chr16:19870688-19870629 | THC2469888 | Hs.148685 |
| A_33_P3246553 | GRIK3 | up | hs\|1p34.3 | 2899 | NM_000831 | chr1:37266697-37266638 | THC2478657 | Hs.128848 |
| A_24_P51201 | HERC3 | down | hs\|4q22.1 | 8916 | BC038960 | chr4:89578269-89578328 | THC2472353 | Hs.35804 |
| A_23_P120883 | HMOX1 | down | hs\|22q12.3 | 3162 | NM_002133 | chr22:35790122-35790181 | THC2468207 | Hs.517581 |
| A_23_P25030 | HSD17B6 | up | hs\|12q13.3 | 8630 | NM_003725 | chr12:57180933-57180992 | THC2472295 | Hs.524513 |
| A_23_P97990 | HTRA1 | down | hs\|10q26.13 | 5654 | NM_002775 | chr10:124274338-124274397 | NP710487 | Hs.501280 |
| A_23_P13907 | IGF1 | down | hs\|12q23.2 | 3479 | NM_000618 | chr12:102796325-102796266 | THC2640819 | Hs.160562 |
| A_23_P150609 | IGF2 | down | hs\|11p15.5 | 3481 | NM_000612 | chr11:2150453-2150394 | THC2465311 | Hs.272259 |
| A_32_P78101 | IGSF21 | down | hs\|1p36.13 | 84966 | NM_032880 | chr1:18704906-18704965 | THC2464101 | Hs.212511 |
| A_23_P129665 | ITGAD | down | hs\|16p11.2 | 3681 | NM_005353 | chr16:31437568-31437627 | THC2601106 | Hs.679163 |
| A_23_P500353 | KCNN2 | down | hs\|5q22.3 | 3781 | NM_021614 | chr5:113831835-113831894 | THC2788986 | Hs.98280 |
| A_23_P130352 | KCTD1 | up | hs\|18q11.2 | 284252 | NM_198991 | chr18:24056609-24056550 | THC2722589 | Hs.526630 |
| A_33_P3410026 | KIAA1324 | up | hs\|1p13.3 | 57535 |  | chr1:109656340-109656399 |  |  |
| A_23_P25994 | LGMN | down | hs\|14q32.12 | 5641 | NM_001008530 | chr14:93178016-93176171 | NP1188457 | Hs.18069 |
| A_33_P3402943 | LOC100128591 | down | hs\|20q13.12 | 100128591 | AK128705 | chr20:44961061-44961002 | THC2514361 | Hs.640065 |
| A_33_P3327300 | LOC100130713 | up | hs\|19q13.2 | 100130713 | AK096566 | chr19:41191283-41191224 | THC2481047 | Hs.326953 |
| A_32_P65589 | LOC100130811 | down |  | 100130811 | XM_003403830 |  | NP303356 | Hs.727319 |
| A_33_P3416142 | LOC100131234 | up | hs\|1q32.1 | 100131234 | EF413001 | chr1:198868184-198868125 |  | Hs.711077 |
| A_33_P3332329 | LOC120824 | up | hs\|11p11.12 | 120824 | NM_001206625 | chr11:48997363-48997304 | THC2479183 | Hs.326734 |
| A_33_P3527721 | LOC284219 | up | hs\|18p11.22 | 284219 | AK094436 | chr18:8767695-8767754 | THC2615581 | Hs.657510 |
| A_33_P3672756 | LOC284561 | up | hs\|1q21.2 | 284561 | XR_112106 | chr1:147763874-147763933 | THC2601545 | Hs.504540 |
| A_33_P3270926 | LOC389607 | down | hs\|8p23.3 | 389607 | XR_110088 | chr8:607638-607579 | THC2484974 | Hs.679027 |
| A_23_P150198 | LOC440040 | up | hs\|11p11.12 | 440040 | NR_027044 | chr11:49831772-49831831 | THC2477950 | Hs.655746 |
| A_33_P3294966 | LOC554206 | up | hs\|16p12.1 | 554206 | NR_038379 | chr16:25043455-25043514 | THC2480737 | Hs.552959 |
| A_33_P3617190 | LOC651337 | down | hs\|9q34.3 | 651337 | AK124119 | chr9:140671107-140671048 | THC2481572 | Hs.603195 |
| A_23_P146233 | LPL | down | hs\|8p21.3 | 4023 | NM_000237 | chr8:19824187-19824246 | THC2465480 | Hs.180878 |
| A_23_P203888 | MMP19 | down | hs\|12q13.2 | 4327 | NM_002429 | chr12:56230495-56230436 | THC2488331 | Hs.591033 |
| A_23_P12746 | MRC1 | down | hs\|10p12.33 | 4360 | NM_002438 | chr10:18199506-18199565 | THC2464756 | Hs.75182 |
| A_24_P372223 | MSR1 | down | hs\|8p22 | 4481 | NM_138715 | chr8:15965525-15965466 | THC2472840 | Hs.147635 |
| A_33_P3379326 | MSR1 | down | hs\|8p22 | 4481 | NM_138715 | chr8:15967653-15967594 | THC2472840 | Hs.147635 |
| A_23_P206920 | MYH11 | up | hs\|16p13.11 | 4629 | NM_001040114 | chr16:15808796-15797952 | NP1146952 | Hs.460109 |
| A_24_P226116 | NAA15 | up | hs\|4q31.1 | 80155 | NM_057175 | chr4:140311796-140311855 | THC2661399 | Hs.555985 |
| A_33_P3403666 | NDOR1 | down | hs\|9q34.3 | 27158 | NM_001144026 | chr9:140112373-140112432 | THC2626702 | Hs.512564 |
| A_23_P127584 | NNMT | down | hs\|11q23.2 | 4837 | NM_006169 | chr11:114183072-114183131 | THC2636921 | Hs.503911 |
| A_24_P928052 | NRP1 | down | hs\|10p11.22 | 8829 | NM_003873 | chr10:33466995-33466936 | THC2495125 | Hs.131704 |
| A_24_P270728 | NUPR1 | down | hs\|16p11.2 | 26471 | NM_001042483 | chr16:28548880-28548821 | THC2554444 | Hs.513463 |
| A_33_P3405399 | ODF2L | down | hs\|1p22.3 | 57489 | NM_001184765 | chr1:86824530-86824471 | THC2562265 | Hs.149360 |
| A_24_P406601 | OLFM1 | down | hs\|9q34.3 | 10439 | NM_014279 | chr9:138012822-138012881 | THC2467893 | Hs.522484 |
| A_33_P3344454 | OR4F6 | up | hs\|15q26.3 | 390648 | NM_001005326 | chr15:102346802-102346861 | NP1461775 | Hs.553399 |
| A_24_P339944 | PDGFB | down | hs\|22q13.1 | 5155 | NM_002608 | chr22:39620158-39620099 | NP1151998 | Hs.1976 |
| A_33_P3345743 | PFN1P2 | up | hs\|1q21.1 | 767846 | NR_003242 | chr1:144611135-144611076 | THC2478720 | Hs.657186 |
| A_23_P21485 | PID1 | down | hs\|2q36.3 | 55022 | NM_017933 | chr2:229889030-229888971 | THC2606128 | Hs.409352 |
| A_23_P252062 | PPARG | down | hs\|3p25.2 | 5468 | NM_138711 | chr3:12458499-12458558 | THC2604982 | Hs.162646 |
| A_33_P3350726 | PPARG | down | hs\|3p25.2 | 5468 | NM_138711 | chr3:12434128-12434187 | THC2708492 | Hs.162646 |
| A_23_P121596 | PPBP | down | hs\|4q13.3 | 5473 | NM_002704 | chr4:74853358-74853299 | THC2473736 | Hs.2164 |
| A_32_P225816 | PRDM16 | down | hs\|1p36.32 | 63976 | NM_022114 | chr1:3355062-3355121 | THC2601645 | Hs.99500 |
| A_33_P3256695 | PRPF40A | up | hs\|2q23.3 | 55660 | NM_017892 | chr2:153508166-153508107 | THC2658013 | Hs.643580 |
| A_33_P3229918 | PTCRA | down | hs\|6p21.1 | 171558 | NM_001243168 | chr6:42893514-42893573 | THC2488468 | Hs.169002 |
| A_23_P134237 | RARRES2 | down | hs\|7q36.1 | 5919 | NM_002889 | chr7:150037260-150037201 | THC2711667 | Hs.647064 |
| A_33_P3307157 | RBM20 | down | hs\|10q25.2 | 282996 | NM_001134363 | chr10:112543126-112543185 |  | Hs.116630 |
| A_23_P257649 | RBP1 | down | hs\|3q23 | 5947 | NM_002899 | chr3:139257729-139257670 | THC2673000 | Hs.529571 |
| A_33_P3240532 | RGL1 | down | hs\|1q25.3 | 23179 | NM_015149 | chr1:183897605-183897664 | THC2491846 | Hs.497148 |
| A_32_P227605 | RGS9BP | up | hs\|19q13.11 | 388531 | NM_207391 | chr19:33169011-33169070 | THC2478356 | Hs.528491 |
| A_23_P51690 | RHBG | down | hs\|1q22 | 57127 | NM_020407 | chr1:156354895-156354954 | THC2706811 | Hs.131835 |
| A_33_P3358745 | SEPP1 | down | hs\|5p12 | 6414 | NM_001093726 | chr5:42800881-42800822 | THC2523981 | Hs.730647 |
| A_23_P121926 | SEPP1 | down | hs\|5p12 | 6414 | NM_005410 | chr5:42799939-42799904 | THC2498975 | Hs.730647 |
| A_23_P43175 | SEPT10 | down | hs\|2q13 | 151011 | NM_144710 | chr2:110300744-110300685 | NP1467265 | Hs.469615 |
| A_33_P3353916 | SETMAR | up | hs\|3p26.1 | 6419 | NM_006515 | chr3:4354913-4354972 | THC2471661 | Hs.475300 |
| A_24_P934387 | SLC5A12 | down | hs\|11p14.2 | 159963 | NM_178498 | chr11:26689385-26689326 | THC2683274 | Hs.148907 |
| A_23_P357983 | STOML3 | up | hs\|13q13.3 | 161003 | NM_145286 | chr13:39540934-39540875 | THC2477731 | Hs.327794 |
| A_33_P3327063 | STRC | down | hs\|15q15.3 | 161497 | NM_153700 | chr15:43900118-43897597 | THC2624821 | Hs.657395 |
| A_23_P87011 | TAGLN | down | hs\|11q23.3 | 6876 | NM_001001522 | chr11:117075155-117075214 | THC2499202 | Hs.410977 |
| A_23_P87013 | TAGLN | down | hs\|11q23.3 | 6876 | NM_001001522 | chr11:117074574-117074963 | THC2537883 | Hs.410977 |
| A_23_P359746 | TAS2R38 | up | hs\|7q34 | 5726 | NM_176817 | chr7:141672993-141672934 | THC2484052 | Hs.647085 |
| A_23_P151297 | TENC1 | down | hs\|12q13.13 | 23371 | NM_015319 | chr12:53457567-53457626 | NP1133710 | Hs.6147 |
| A_23_P212508 | TF | down | hs\|3q22.1 | 7018 | NM_001063 | chr3:133495990-133496049 | THC2533500 | Hs.518267 |
| A_23_P168329 | TINAG | down | hs\|6p12.1 | 27283 | NM_014464 | chr6:54214641-54216155 | NP186271 | Hs.127011 |
| A_33_P3395605 | TMEM119 | down | hs\|12q23.3 | 338773 | NM_181724 | chr12:108983690-108983631 | THC2484324 | Hs.449718 |
| A_33_P3289296 | TMEM37 | down | hs\|2q14.2 | 140738 | NM_183240 | chr2:120196037-120196096 |  | Hs.26216 |
| A_23_P48803 | TMOD2 | down | hs\|15q21.2 | 29767 | NM_014548 | chr15:52101182-52101241 | THC2473231 | Hs.513734 |
| A_33_P3381777 | TREML1 | down | hs\|6p21.1 | 340205 | NM_178174 | chr6:41117457-41117398 | NP1465593 | Hs.117331 |
| A_23_P64306 | TRIM48 | up | hs\|11q11 | 79097 | NM_024114 | chr11:55032550-55032609 | THC2477950 | Hs.195715 |
| A_23_P1575 | TRIM49 | up | hs\|11q14.3 | 57093 | NM_020358 | chr11:89531619-89531560 | THC2481696 | Hs.534218 |
| A_33_P3395581 | TRIM53P | up | hs\|11q14.3 | 642569 | NR_028346 | chr11:89727098-89727044 |  |  |
| A_33_P3310744 | TTC34 | down | hs\|1p36.32 | 100287898 | NM_001242672 | chr1:2572866-2572807 |  | Hs.632363 |
| A_33_P3259625 | TTC34 | down | hs\|1p36.32 | 100287898 | NM_001242672 | chr1:2700249-2700190 |  | Hs.632363 |
| A_23_P55356 | VMO1 | down | hs\|17p13.2 | 284013 | NM_182566 | chr17:4688732-4688673 | THC2477391 | Hs.122561 |

**SUPPLEMENTAL FIGURES**


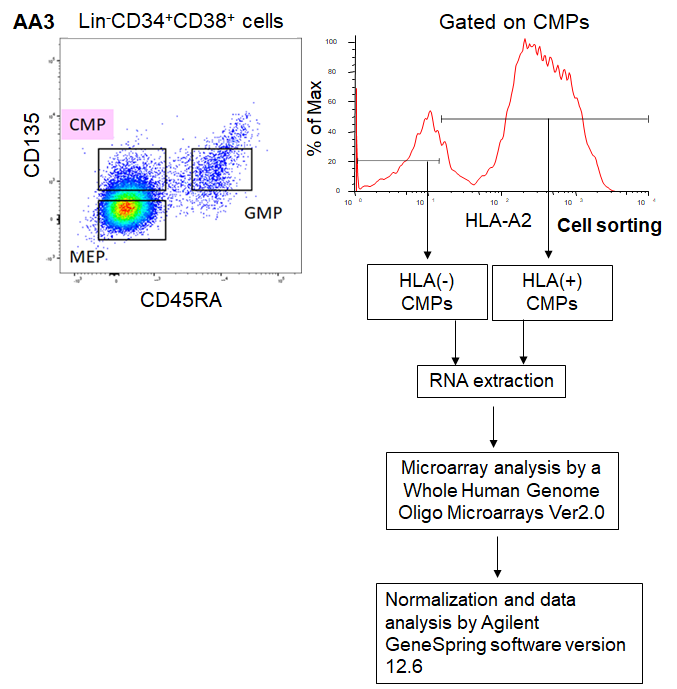
**Figure S1. A work flow to determine the gene expression profile by CMPs from AA patients with microarray analysis**


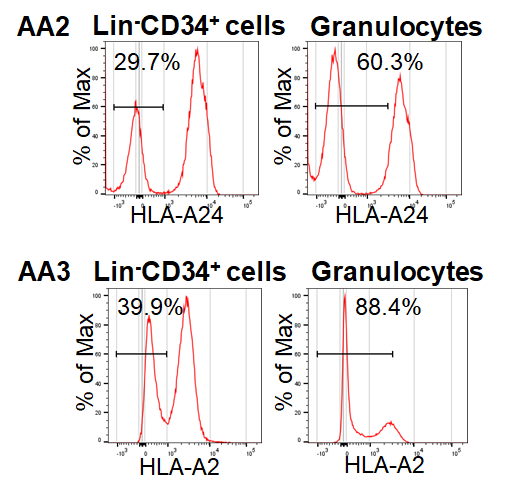
**Figure S2. The percentage of HLA(-) cells in CD34^+^ cells of BM and granulocytes from AA patients**

Histograms of HLA(-) cells in Lin^-^CD34^+^ cells and granulocytes from two AA patients (AA2 and AA3) are shown.


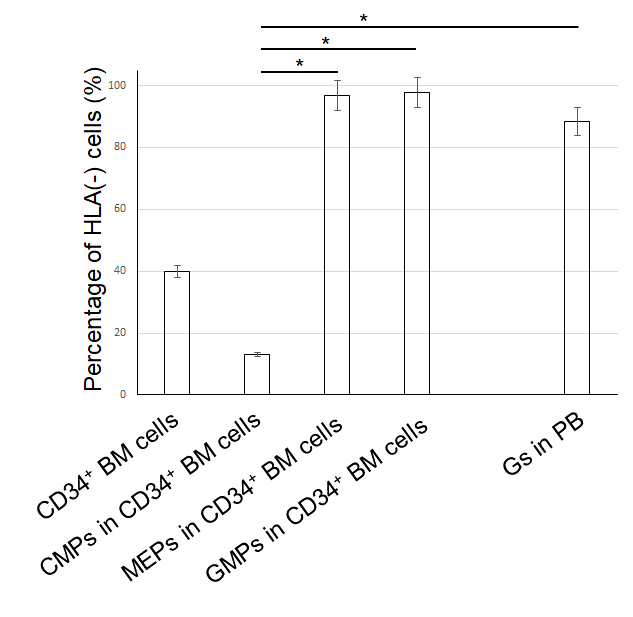
**Figure S3. The percentage of HLA(-) cells in CD34^+^ subsets of BM and granulocytes in PB from three AA patients**

The bar graph summarizes the percentages of HLA(-) cells in CD34^+^ subsets of BM and granulocytes in PB from three AA patients (AA1-3). **P*<0.05. CMPs, common myeloid progenitors; MEPs, myeloid-erythroid progenitors; GMPs, granulocyte-macrophage progenitors; Gs, granulocytes.

**REFERENCES**

1. Camitta BM, Rappeport JM, Parkman R, Nathan DG. Selection of patients for bone marrow transplantation in severe aplastic anemia. *Blood*. 1975;45(3):355-63.

2. Bacigalupo A, Hows J, Gluckman E, Nissen C, Marsh J, Van Lint MT, et al. Bone marrow transplantation (BMT) versus immunosuppression for the treatment of severe aplastic anaemia (SAA): a report of the EBMT SAA working party. *Br J Haematol*. 1988;70(2):177-82.

3. Camitta BM, Doney K. Immunosuppressive therapy for aplastic anemia: indications, agents, mechanisms, and results. *Am J Pediatr Hematol Oncol*. 1990;12(4):411-24.

4. Katagiri T, Sato-Otsubo A, Kashiwase K, Morishima S, Sato Y, Mori Y, et al. Frequent loss of HLA alleles associated with copy number-neutral 6pLOH in acquired aplastic anemia. *Blood*. 2011;118(25):6601-9.

5. Arber DA, Orazi A, Hasserjian R, Thiele J, Borowitz MJ, Le Beau MM, et al. The 2016 revision to the World Health Organization classification of myeloid neoplasms and acute leukemia. *Blood*. 2016;127(20):2391-405.

6. Cheson BD, Greenberg PL, Bennett JM, Lowenberg B, Wijermans PW, Nimer SD, et al. Clinical application and proposal for modification of the International Working Group (IWG) response criteria in myelodysplasia. *Blood*. 2006;108(2):419-25.

7. Doulatov S, Notta F, Eppert K, Nguyen LT, Ohashi PS, Dick JE. Revised map of the human progenitor hierarchy shows the origin of macrophages and dendritic cells in early lymphoid development. *Nat Immunol*. 2010;11(7):585-93.

8. Maruyama H, Katagiri T, Kashiwase K, Shiina T, Sato-Otsubo A, Zaimoku Y, et al. Clinical significance and origin of leukocytes that lack HLA-A allele expression in patients with acquired aplastic anemia. *Exp Hematol*. 2016;44(10):931-9 e3.

9. Tusher VG, Tibshirani R, Chu G. Significance analysis of microarrays applied to the ionizing radiation response. *Proc Natl Acad Sci U S A*. 2001;98(9):5116-21.
